# Detection of Clones B2-ST131-C2 and A-ST617 in *Escherichia coli* Producing Both CTX-M-15 and CTX-M-27 from Tunisian Community Patients

**DOI:** 10.3390/antibiotics11101329

**Published:** 2022-09-29

**Authors:** Amel Mhaya, Rahma Trabelsi, Sabine Aillerie, Fatima M’Zali, Dominique Bégu, Slim Tounsi, Radhouane Gdoura, Corinne Arpin

**Affiliations:** 1 University of Bordeaux, Department of Biological and Medical Sciences, UMR CNRS 5234, 146 Rue Léo Saignat, 33076 Bordeaux, France; 2 Centre of Biotechnology of Sfax, Laboratory of Biopesticides, Road of Sidi Mansour Km 6, 3018 Sfax, Tunisia; 3 University of Sfax, Department of Life Science, Research Laboratory of Environmental Toxicology-Microbiology and Health, Road of Soukra Km 3.5, 3000 Sfax, Tunisia; 4 University of Bordeaux, Aquitaine Microbiologie, 146 Rue Léo Saignat, 33076 Bordeaux, France

**Keywords:** CTX-M-15, CTX-M-27, *Escherichia coli*, ST131, ST617, plasmid-mediated resistance

## Abstract

During a two-month period (2017–2018), 336 urine samples positive for *Escherichia coli* were collected from Tunisian patients. Of the 336 samples, 266 were collected from community patients and 70 from hospital settings. In all, 15 ESBL producers were identified (8 and 7, respectively) and assigned to 13 pulsotypes, including four ESBL-producing *E. coli* (ESBL-E) with E1 and E2 profiles (2 isolates each) from community patients. The two strains E1 were identified as B2-ST131 subclade C2 and the two isolates E2, A-ST617. The four strains carrying both CTX-M-15 and CTX-M-27, exhibited the multireplicon IncFII/F1A/F1B with the same formula F31:A4:B1. Two isolates with patterns E3 and E4 (Dice coefficient, 78.7%) isolated from community and hospital settings of two geographic areas were assigned to the emerging ST131 C1-M27 subclade and contained the replicon F1:A-:B20. The remaining ESBL-E divided into different sequence types/associated CTX-M: 2 ST131-C2/CTX-M-15 and ST744/CTX-M-55, ST617/CTM-15, ST2973/CTX-M-55, ST6448/CTX-M-15, ST224/CTX-M-15, ST1431/CTX-M-15, and ST38/CTX-M-27, one isolate each. Our study reports for the first time the presence in the Tunisian community of two clones of *E. coli*, including the virulent clone ST131-C2 harboring both CTX-M-15 and CTX-M-27, and confirms the spread of the emergent clone ST131-C1-M-27, notably in community urinary tract infections.

## 1. Introduction

Resistance to extended-spectrum cephalosporins is widespread among the *Enterobacterales* species and is mostly due to the dissemination of extended-spectrum ß-lactamases (ESBLs), which are becoming a major global public health concern [1,2]. Initially reported in the late 1980s, CTX-M-type ESBLs (CTX-M for CefoTaXimase from Munich) have emerged since the 2000s, and are now considered as pandemic enzymes [1,3]. Many *bla*_CTX-M_ variants exist and most of them can be clustered into five groups based on sequence homologies; each cluster of CTX-M genotypes has a corresponding progenitor gene sharing homology with different environmental *Kluyvera* spp., from which the *bla*_CTX-M_ genes originated [1,3]. In clinical isolates and by far, the most prevalent in the CTX-M-1 group is CTX-M-15. In the CTX-M-9 group, CTX-M-9 and CTX-M-14 are the most common enzymes, but recent surveillance studies showed that CTX-M-27 is emerging in different parts of the world [1,3]. The role of mobile genetic elements and horizontal gene transfer in the dissemination of the *bla*_CTX-M_ genes has been well documented [4]. The *bla*_CTX-M_ genes are often present on conjugative plasmids belonging to the IncF incompatibility group, which may be multireplicons harboring IncFII, IncFIA and IncIB. These conjugative plasmids can also carry other resistance genes, thus playing a major role in the spread of multidrug resistance [4,5]. 

*Escherichia coli* is the predominant bacteria found in human infections, mainly in urinary tract infections (UTI). It is the most prevalent causative agent found associated with global ESBLs [3]. CTX-M-producing *E. coli* associated with UTI are often encountered within specific lineage belonging to the highly virulent phylogenetic group B2 of the sequence type (ST) 131 [6]. This high-risk ST131 lineage has been disseminated worldwide with sublineages classified into three clades (A, B, and C) and different C subclades [7]. Phylogenetic studies have revealed a frequent association of CTX-M-15 with the subclade C2 [8]. More recently, a C1 subclade associated with the *bla*_CTX-M-27_ gene reported as C1-M27 was detected in Japan, and rapidly emerged in different parts of the world [1,9], including North Africa [10,11].

In Tunisia, the presence of ESBL-producing *E. coli* (ESBL-E) has been increasingly reported in community-acquired UTI since 2013 [12,13]. In the present work, during a two-month period, urine samples were collected from patients of two private biological laboratories situated at Sfax. With the aim of comparing their possible epidemiological relationships during the same period, urine samples of patients from healthcare facilities of two distant Tunisian locations (Sfax and Tunis) were also included in the study. 

In this study, 15 ESBL-E (eight in community and seven in hospital) were characterized with regard to their ESBL and plasmid contents. Their clonal epidemiological relationships were analyzed by phylogroup, pulsotype and sequence-type determination.

## 2. Results

### 2.1. Prevalence of ESBL-Producing E. coli

Between December 2017 and January 2018, 266 non-redundant *E. coli* strains were collected from the urine samples of community patients (one isolate per patient) divided into 60 and 206 from two private laboratories, CA and CB respectively. Among them, eight isolates produced ESBL as confirmed by phenotypic and molecular characterizations (see Section 4. Materials and Methods), giving a prevalence rate of 1.7% (in CA) and 3.4% (in CB). 

During the same period, 70 non-repetitive isolates of *E. coli* were randomly recovered among a collection of urine positive cultures from three healthcare facilities. One of them (polyclinic, H1) situated at Sfax provided samples mainly collected from patients of a urology unit. The two others (H2, regional hospital and H3, psychiatric clinic) were located in Tunis; both locations (Sfax and Tunis) are ca. 270-km away. Among them, seven ESBL producers were isolated, giving an overall prevalence rate of 10% divided into 7.5% in H1 (3 among 40 isolates), and 20% (3/15) and 6.7% (1/15) in H2 and H3, respectively. Thus, a total of 15 ESBL-producing *E. coli* (ESBL-E) were recovered in our study.

### 2.2. Analysis of β-Lactamase Content

All ESBL-E gave positive PCR amplifications with specific primers for the *bla*_CTX-M_ genes. Surprisingly, four isolates contained two groups of CTX-M: CTX-M-15 (group 1) and CTX-M-27 (group 9). Five *E. coli* carried CTX-M-15, three CTX-M-55, and the three remaining carried CTX-M-27. Six and four isolates also exhibited OXA-1-like and TEM-1-like ß-lactamases, respectively, including one that carried both enzymes. No other ß-lactamases genes screened (plasmid-mediated cephalosporinase and carbapenemase genes) were identified. 

### 2.3. Molecular Typing Analysis 

**Figure 1:**
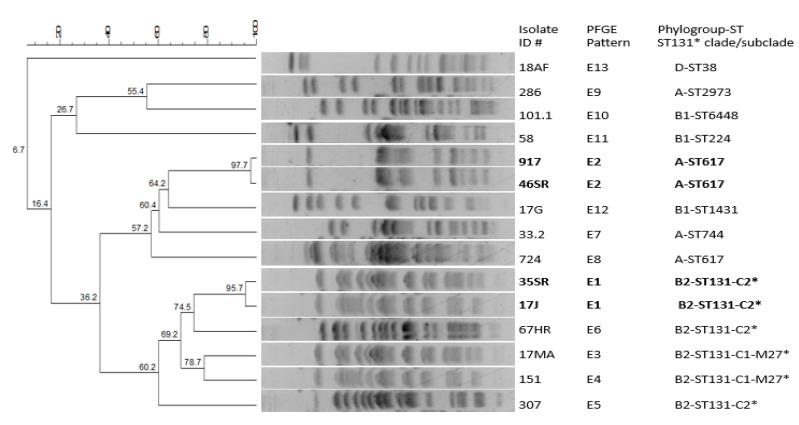
Dendogram based on pulsotypes after *Xba*I digestion of 15 CTX-M-producing *E. coli.* IDentification number (ID#) of isolates, PFGE pattern, phylogroup, ST and for ST131*, clade/subclade are indicated. The four isolates with both CTX-M-25 and CTX-M-27 are shown in bold.

The ESBL-E mainly belonged to phylogenetic group B2 (40%), followed by the groups A (33.3%), B1 (20%) and D (6.7%) (Table 1). The 15 *E. coli* were assigned to 13 distinct PFGE patterns (pulsotypes E1 to E13, Figure 1 and Table 1). The four ESBL-E with both CTX-M-15 and CTX-M-27 were divided into two isolates (IDentification number, ID# 35SR and 17J) of pattern E1 (Dice coefficient, 95.7%), belonging to clone B2-ST131 subclade C2, and two isolates (ID# 917 and 46SR) of profile E2 (Dice coefficient, 97.7%), which were ascribed to A-ST617. Two isolates (ID# 17MA and 151) with CTX-M-27 of patterns E3 and E4 (Dice coefficient, 78.7%), were assigned to B2-ST131 subclade-C1-M27. The remaining ESBL-E were obtained as following (phylogroup/ST/associated CTX-M): two isolates of B2-ST131-C2/CTX-M-15, and one isolate each of A-ST744/CTX-M-55, A-ST617/CTM-15, A-ST2973/CTX-M-55, B1-ST6448/CTX-M-15, B1-ST224/CTX-M-15, B1-ST1431/CTX-M-15, ST38/CTX-M-27 (Figure 1 and Table 1).

### 2.4. Antibiotic Resistance Pattern

**Figure 2:**
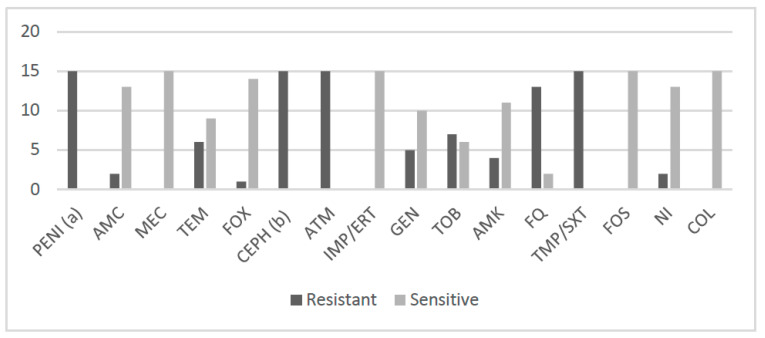
Proportion of antibiotic resistances among the 15 ESBL-E. PENI (a), ampicillin, ticarcillin, piperacillin; AMC, amoxicillin-clavulanic acid; MEC, mecillinam; TEM, temocillin (susceptible with increased exposure); FOX, cefoxitin; CEPH (b), cefixime, cefuroxime, ceftriaxone, ceftazidime, cefepime; ATM, aztreonam; IMP/ERT, imipenem/ertapenem; GEN, gentamicin; TOB, tobramycin; AMK, amikacin; FQ, fluoroquinolones (ciprofloxacin, ofloxacin); TMP/SXT, trimethoprim/sulfamethoxazole-trimethoprim; FOS, fosfomycin; NI, nitrofurantoin; COL, colistin.

The double-disk synergy test was positive for the 15 isolates. All CTX-M-producing *E. coli* remained susceptible to carbapenems and only one strain (ID# 67HR) exhibited a resistance to cefoxitin (cephamycin), which could be attributed to an outer membrane impermeability or to overexpression of the chromosomal cephalosporinase. Cefotaxime MICs values were high (≥1024 µg/mL) except for two isolates (ID# 17MA and 151) with MICs of 256 µg/mL. Ceftazidime MICs ranged from 4 to 64 µg/mL (Table 1). The ESBL-E also exhibited resistances to trimethoprim and cotrimoxazole (100%), fluoroquinolones (87%) and to aminoglycosides as follows: gentamicin, tobramycin and amikacin, 33%, 47% and 27%, respectively. All remained susceptible to fosfomycin and colistin (≤2 µg/mL), and two isolates (ID# 917 and 46SR) were resistant to nitrofurantoin (Table 1, Figure 2 and Table S1 in Supplementary Material).

### 2.5. Plasmid Content Analysis 

ESBL-E contained plasmids that were assigned to the IncF replicon. IncFII was predominantly found (*n* = 11), both with IncFIA (*n* = 5) and IncFIB (*n* = 10). The four remaining strains harbored only IncFIB. The IncF replicons were not always associated with the others, IncN, IncY, IncW and IncX (Table 1). Four isolates of lineages ST131-C2 and ST617 contained a multireplicon IncFII/FIA/FIB with the formula: F31:A4:B1. The 2 *E. coli* ST131 C1-M27 contained the replicon F1:A-B20.

## 3. Discussion

In Tunisia, investigations of ESBL-producing bacteria in community-infected patients are rare. In our study, the rate of ESBL-producing *E. coli* among UTI in the community reached 3.4%, and it is consistent with recent data [13], confirming that the proportion of such strains has increased in this country since 2013 [12]. Higher prevalence rates were found in healthcare facilities. The clinical data associated with the acquisition of these bacteria were not collected in this study, but previous studies reported risk factors including length of hospitalization, presence of an indwelling urinary catheter, prior exposure to antibiotics and underlying illness [16]. According to the Laboratory of Antibio-Resistance in Tunisia (LART), which is a national hospital surveillance system, the global rate of *E. coli* resistance to broad-spectrum cephalosporins with a positive synergy test was 17.4 % in 2017 (10.7 to 30.7%, depending on the hospital) [17].

The main finding of our work is the detection of two clones of *E. coli* producing concomitantly two groups of CTX-M. In the literature, very few studies mentioned the presence of such strains, and only with a low proportion in the ST131 lineage [18,19,20] and, to our knowledge, there is to date no report from Tunisia. The virulent ST131-C2 *E. coli* is a predominant lineage among extraintestinal pathogenic *E. coli* (ExPEC), which has played a major role in the worldwide dissemination of CTX-M-15 [6]. This pandemic clone has been reported in human beings but also from non-human sources including animals, the food chain and the environment in Tunisia [11,21,22,23], as well as in Algeria, a neighboring country [24]. *E. coli* A-ST617 related to the clonal complex 10 (Cplx10) is less often encountered among the CTX-M-15-producing non-ST131 isolates. However, it has emerged in Africa (Nigeria) since the beginning of the 2010s [25]. It has also been found in 2015 from a colonized patient newly admitted in a hospital of Tunis [23]. A systematic review conducted among ExPEC lineages reported proportions for ST617 higher from Africa than studies from Asia and Europe [26]. To the best of our knowledge, we report for the first time in Tunisia, the presence and the spread among four community-infected patients of two multidrug-resistant *E. coli* clones of pulsotypes E1 (ST131-C2) and E2 (A-ST617), carrying not only CTX-M-15 but also the additional CTX-M-27. In our study, three other isolates with only CTX-M-15 belonging to lineages ST131-C2 and A-ST617 were detected in hospital, but their pulsotypes showed no recent clonal relationship with the clones E1 and E2. 

We also describe the presence of subclade ST131-C1-M-27 among Tunisian infected patients (from community and hospital). This clone was described in 2013–2015 in wastewater in Tunisia [11]. Recently, it has been reported with a rate of intestinal carriage of 9% in Tunisian food handlers, and probably with a similar rate among the general population [10]. This presence in both hospital and non-hospital settings, and in different regions, clearly shows the increasing dissemination of this clone in this country, but it remains little reported elsewhere in Africa. 

With regard to the remaining lineages of ESBL-E detected, this study showed a diversity of ESBL and ST. Firstly, D-ST38 (Cplx38) is a widely distributed lineage in association with *bla*_CTX-M-15_ and *bla*_CTX-M-14_ [27]. However, the presence of *bla*_CTX-M-27_ in ST38, as reported in our survey, is less frequent. This association was described in the United States [28], and also in Tunisian wastewater [29] and other African countries [30]. CTX-M-15 is the predominant ESBL described in Tunisia, as also reported in two other phylogroup B1 clones, ST224 and ST1431. *E. coli* strains belonging to B1-ST224/CTX-M-15 are mainly identified in animals [31], but also from clinical isolates of a tertiary hospital in Tanzania [32]. ST1431/CTX-M-15 was described from wild boar in Algeria [33]. In our study, three ST: i.e., A-ST744 (ST10 Cplx), A-ST2973 and B1-ST6448, were associated with the CTX-M-55 variant. This gene first detected in Thailand in 2005 [34] has successfully disseminated in China and worldwide [1,35]. *E. coli* ST744 harboring the *bla*_CTX-M-1_ group has been sporadically described in human beings, but is more commonly found in animal sources in various parts of the world [36], and has also been reported in association with a colistin resistance gene in a French veterinary study [36]. A-ST2973/CTX-M-55 and B1-ST6448/CTX-M-55 has been sporadically isolated in Tunisia from river water and wastewater [29,37]. 

Most of ESBL-E found in our study were resistant to different antibiotic families. In the literature, ESBL enzymes are often identified in multidrug-resistant strains [1]. A linkage between the clone ST131-C and quinolone resistance has also been well documented [6,7]. The CTX-M-15 and CTX-M-27 are ESBL variants that contain a residue change in position 240 (Asp to Gly) conferring higher level resistance to ceftazidime [38]. Although ceftazidime MICs did not show higher values in the two clones with both enzymes compared to other strains with a single ESBL, their concomitant presence may have given them a selective advantage and favored their selection and dissemination under antibiotic pressure.

The *bla*_CTX-M_ genes are largely mediated by conjugative plasmids in *Enterobacterales* [5,39]. Plasmids, especially those belonging to the incompatibility group IncFII, additionally harboring FIA/FIB replicons, have been described as contributing to the successful spread of some pandemic clones, including the major CTX-M-15-producing *E. coli* ST131 [8,40]. In our study, the lineages B2-ST131-C2 and A-ST167 harboring *bla*_CTX-M-15_ and *bla*_CTX-M-27_ genes contained a multireplicon IncFII/FIA/FIB with the same combination (FII:FIA:FIB), i.e., F31:A4:B1, suggesting a possible horizontal transfer. In the literature, this F31:A4:B1 replicon carrying only the *bla*_CTX-M-15_ gene has been identified in ST131-C2, and also ST617 isolates in different countries worldwide, including Tunisia [15,41]. Since no other common replicons were found in these two clones, we hypothesize the possible additional acquisition of the *bla*_CTX-M-27_ gene in this replicon. Investigations are needed to characterize it further and to verify whether the acquisition of the *bla*_CTX-M-27_ gene is due to independent events. The two *E. coli* ST131 C1-M27 contained the replicon F1:A-B20, and in the literature the F1:A2:B20 plasmid is often associated with the C1-M-27 clade isolates [42], but the replicon F1:A-B20 without FIA is poorly documented. However, its presence in strains found in different geographical areas (Tunis and Sfax) could suggest that it is well established in Tunisia. 

## 4. Materials and Methods

### 4.1. Strains Collection and Identification of E. coli

Over the study period, a total of 646 urine samples were recovered from community patients from two private biological Laboratories in Sfax (Laboratories CA and CB). During the same period, of the 846 urine positive cultures collected from patients of three healthcare facilities, 200 were randomly selected. The samples originated from a polyclinic located in Sfax (named H1) and from two hospitals in Tunis (H2 and H3). The identification of *E. coli* was firstly carried out by using the API^®^ 10S strip method (BioMerieux) and confirmed by using the Matrix-Assisted Laser Desorption Ionization-Time-Of-Flight/Mass Spectrometry (MALDI-TOF/MS, Brüker Daltonics), as previously described [43].

### 4.2. Antibiotic Susceptibility Testing

Antibiotic susceptibility patterns of *E. coli* were performed by the agar diffusion method according to the recommendations of the European Committee on Antimicrobial Susceptibility Testing (EUCAST) [44]. The antibiotic disks (Bio-Rad) used were the following: ampicillin, amoxicillin-clavulanic acid, ticarcillin, ticarcillin-clavulanic acid, piperacillin, piperacillin-tazobactam, mecillinam, temocillin, cefoxitin, cefixime, cefuroxime, ceftriaxone, ceftazidime, cefepime, aztreonam, imipenem, ertapenem, gentamicin, tobramycin, amikacin, acid nalixidic, ciprofloxacin, ofloxacin, cotrimoxazole, trimethoprim, fosfomycin, nitrofurantoin. Isolates were screened for ESBL production by using the double-disk synergy test. MICs of cefotaxime, ceftazidime and colistin were determined by the micro-dilution method. Results were interpreted according to EUCAST breakpoints as updated in 2022 [44].

### 4.3. Molecular Characterization of ß-Lactamase Genes

After extraction, total DNA of all the broad-spectrum cephalosporin-resistant *E. coli* was screened for the ß-lactamases presence, which included multiplex PCR by amplifications for types TEM-, SHV- and OXA-1-like enzymes and multiplex PCR for groups -1, -2, -9, -18, -25 CTX-M ß-lactamases, as described by Dallenne et al. [45]. Multiplex PCR designed in the procedure of Dallenne et al. [45] also included the detection of plasmid-mediated AmpC β-lactamases (i.e., ACC, FOX, MOX/CMY, DHA, LAT, ACT/MIR), as well as other important β-lactamases such as carbapenemase genes (*bla*_VIM_, *bla*_IMP_, *bla*_KPC_, *bla*_GES_ and *bla*_OXA-48-like_). Amplifications of entire genes for *bla*_CTX-M-_group 1 (band size, 922 bp) and *bla*_CTX-M_ group 9 (band size, 876 bp) genes were conducted using the following primer pairs: CTXM1F (5′-CTATTCATGTTGTTGTTATTTCG-3′) and CTX-M1R (5′-TTACAAACCGTTGGTGACGA-3′) and CTXM9F (5′-ATGGTGACAAAGAGAGTGCA-3′) and CTXM9R (5′-TTACAGCCCTTCGGCGATGA-3′). The amplifications using the GoTaq^®^ DNA Polymerase (Promega) were carried out with an initial denaturation at 94 °C for 5 min, followed by 35 cycles of 94 °C for 1 min, 55 °C for 1 min, and 72 °C for 1 min, and final extension at 72 °C for 10 min. The PCR products were sequenced par Eurofins Genomics (Germany GmBH). The sequences were analyzed using the Basic Local Alignment Search Tool (BLAST) [46].

### 4.4. Molecular Typing of ESBL-Producing E. coli 

The clonal relationship among ESBL-E was investigated after *Xba*I enzyme digestion and pulsed-field gel electrophoresis (PFGE) analysis using the CHEF DRII^®^ apparatus (Bio-Rad), as detailed elsewhere [47]. The PFGE patterns were first compared after visual inspection, then a dendrogram was constructed using the DICE similarity coefficient by the UPGMA method (1% tolerance limit and 1% optimization) performed with the software package Bionumerics^®^ 6.3 (Applied Maths). 

ESBL-E were classified into different phylogenetic groups using the triplex PCR (*chuA, yjaA* and *TSPE4.C2*) [48]. Multilocus Sequence Typing (MLST) consisted of the genotyping of seven house-keeping genes (*adk*, *fumC*, *gyrB*, *icd*, *mdh*, *purA*, *recA*) as described in the website from the Achtman scheme [49,50]. Furthermore, we used the specific PCR procedure described by Matsumura et al. [14], for clades and subclades assignation of *E. coli* ST131. 

### 4.5. Plasmid Analysis

The identification of group incompatibility plasmids were subjected to PCR-based replicon typing (PBRT) method [51]. The presence of IncFII, IncFIA, IncFIB were characterized using the PCR amplification procedure of Villa et al. [15], and after sequencing, the IncF replicon typing scheme was assigned to the FAB formula by using the web tool described by Carattoli et al. [39].

## 5. Conclusions

In this study, we identified, for the first time, in Tunisia and in community-acquired infections, the presence of both CTX-M-15 and M-27 in at least two clones of E. coli including the virulent clone, ST131-C2. The two clones share a common plasmid F31:A4:B1, which may have contributed to the spread of these two ESBLs which are known to confer high level resistances to extended-spectrum cephalosporins. This study also reported the presence of subclade ST131-C1-M-27 among Tunisian community-infected patients and confirmed its implantation in the country. Overall, our results highlight the need for a sustained monitoring and reinforcement of hygiene measures to prevent human transmission and the spread of multi-drug resistant bacteria not only in hospital but also in the community setting in Tunisia.

## Figures and Tables

**Table 1:** Characteristics of the 15 ESBL-producing *E. coli*.

Isolate ID # ^(a)^	Location ^(b)^	ESBL Content	Other ß-Lactamase	MIC (µg/mL) ^(c)^		Associated Resistances ^(c)^	PFGE Profile	Phylogroup-ST ST131 Subclade ^(d)^	Inc Plasmid pMLST ^(e)^
				CAZ	CTX				
**35SR**	**Sfax/CB**	**CTX-M-15 CTX-M-27**	**OXA-1-like**	16	**2,048**	**FQ GEN TOB AMK SXT**	**E1**	**B2-ST131-C2 ^(d)^**	**F31:A4:B1 ^(e)^/W**
**17J**	**Sfax/CB**	**CTX-M-15 CTX-M-27**	**OXA-1-like**	16	**2,048**	**FQ TOB AMK SXT**	**E1**	**B2-ST131-C2 ^(d)^**	**F31:A4:B1 ^(e)^**
17MA	Sfax/CB	CTX-M-27	-	8	256	FQ SXT	E3	B2-ST131-C1-M27 ^(d)^	F1:A-:B20 ^(e)^
151	Tunis/H2	CTX-M-27	-	8	256	FQ SXT	E4	B2-ST131-C1-M27 ^(d)^	F1:A-:B20 ^(e)^
307	Tunis/H2	CTX-M-15	TEM- and OXA-1-like	32	1,024	FQ FOX GEN TOB AMK SXT	E5	B2-ST131-C2 ^(d)^	FII:FIA
67HR	Tunis/H3	CTX-M-15	OXA-1-like	64	4,096	FQ TOB AMK SXT	E6	B2-ST131-C2 ^(d)^	FII:FIB/Y
**917**	**Sfax/CB**	**CTX-M-15 CTX-M-27**	**OXA-1-like**	16	**1,024**	**FQ GEN TOB SXT NI**	**E2**	**A-ST617**	**F31:A4:B1 ^(e)^/N**
**46SR**	**Sfax/CB**	**CTX-M-15 CTX-M-27**	**OXA-1-like**	32	**1,024**	**FQ GEN TOB SXT NI**	**E2**	**A-ST617**	**F31:A4:B1 ^(e)^/N**
33.2	Sfax/H1	CTX-M-55	TEM-like	16	4,096	FQ GEN TOB SXT	E7	A-ST744	FII:FIB/W/X
724	Tunis/H1	CTX-M-15	-	64	2,048	FQ SXT	E8	A-ST617	FII: FIB/Y
286	Tunis/H2	CTX-M-55	-	16	4,096	FQ SXT	E9	A-ST2973	FII:FIB/Y
101.1	Sfax/CA	CTX-M-55	-	16	4,096	FQ SXT	E10	B1-ST6448	FIB
58	Sfax/H1	CTX-M-15	TEM-like	8	1,024	SXT	E11	B1-ST224	FIB/W/N/Y
17G	Sfax/CB	CTX-M-15	TEM-like	8	1,024	FQ SXT	E12	B1-ST1431	FIB/W/N/Y
18AF	Sfax/CB	CTX-M-27	-	4	1,024	SXT	E13	D-ST38	FIB

^(a)^ ID #, identification number of isolates, with those of pulsotypes E1 and E2 indicated in bold. ^(b)^ C, community and H, Healthcare facility (hospital and clinic) settings. ^(c)^ CAZ, ceftazidime; CTX, cefotaxime, FQ, fluoroquinolones (ciprofloxacin and ofloxacin); FOX, cefoxitin; GEN, gentamicin; TOB, tobramycin; AMK, amikacin; SXT, cotrimoxazole; NI, nitrofurantoin. ^(d)^ ST131 clades/subclades were determined according to the procedure of Matsumura et al. [14]. ^(e)^ pMLST of IncF plasmids were performed according to the procedure of Villa et al. [15].

## Supplementary Materials

The following are available online at https://www.mdpi.com/article/10.3390/antibiotics11101329/s1, Table S1: Antibiotic susceptibility testing results of 15 ESBL-E.
