# Supplementary material for: Detection of Clones B2-ST131-C2 and A-ST617 in Escherichia coli Producing Both CTX-M-15 and CTX-M-27 from Tunisian Community Patients
Source: Antibiotics (Basel). 2022 Sep 29;11(10):1329. doi: 10.3390/antibiotics11101329 (PMC9598323; doi:10.3390/antibiotics11101329)
Supplement: Supplementary file 1 [file antibiotics-11-01329-s001.zip › antibiotics-1861103-supplementary.pdf]

Supplementary Material

Table S1. Antibiotic susceptibility testing results of 15 ESBL-E.

Isolate	Diameter of inhibition growth zone (mm) for β-lactam antibiotics: [(a)]																
ID#	AMP	AMC	TIC	TCC	PIP	PZP	MEC	TEM	FOX	CFM	CMX	CRO	CAZ	FEP	ATM	IMP	ERT
35SR	6	16	6	16	6	28	32	11	24	6	6	10	14	20	16	34	30
17J	6	16	6	16	6	28	32	11	24	6	6	10	14	20	16	34	30
17MA	6	22	6	18	10	28	30	20	24	8	6	13	16	22	20	32	28
151	6	24	6	18	8	28	32	20	24	8	8	12	16	20	18	32	30
307	6	13	6	12	6	24	34	8	18	6	6	8	8	14	12	30	28
67HR	6	16	6	14	6	20	30	20	26	6	6	8	10	16	14	30	28
917	6	16	6	16	6	26	34	8	26	6	6	12	14	20	18	34	28
46SR	6	11	6	14	6	26	32	8	22	6	6	8	12	16	14	32	28
33.2	6	16	6	18	6	28	30	14	22	6	6	10	12	16	16	30	28
724	6	22	6	15	6	28	30	18	24	6	6	8	12	16	16	30	28
286	6	22	6	16	6	25	29	22	24	6	6	8	10	14	14	30	25
101.1	6	22	6	16	6	25	30	22	24	6	6	8	10	14	14	30	24
58	6	20	6	18	6	28	30	20	24	8	6	12	14	20	18	36	30
17G	6	20	6	16	6	22	30	18	26	6	6	10	15	18	15	30	28
18AF	6	24	6	20	6	28	32	20	26	6	6	10	16	20	18	32	28

[(a)]) The antibiotic panel included (µg/disk): AMP, ampicillin (10); AMC, amoxicillin-clavulanic acid (20-10); TIC, ticarcillin (75); TCC, ticarcillin-clavulanic acid (75-10); PIP, piperacillin (30); PZP, piperacillin-tazobactam (30-6); MEC, mecillinam (10); TEM, temocillin (30); FOX, cefoxitin (30); CFM, cefixime (5); CMX, cefuroxime (30); CRO, ceftriaxone (30); CAZ, ceftazidime (10); FEP, cefepime (30); ATM, aztreonam (30); IMP, imipenem (10); ERT, ertapenem (10).

Table S1 (continued): Antibiotic susceptibility testing results of 15 ESBL-E.

Isolate	Diameter of inhibition growth zone (mm) for antibiotics other than β-lactams: [(b)]									
ID#	GEN	TOB	AMK	NAL	CIP	OFX	SXT	TMP	FOS	NI
35SR	16	11	17	6	6	6	6	6	30	26
17J	25	11	17	6	6	6	6	6	30	25
17MA	22	22	21	6	6	6	6	6	29	24
151	21	22	24	6	6	6	8	6	28	24
307	10	15	17	6	6	6	6	6	28	26
67HR	20	7	16	6	6	6	6	6	28	20
917	12	11	22	6	8	8	8	6	30	10
46SR	8	10	20	6	6	6	6	6	26	10
33.2	8	11	28	6	6	6	6	6	30	20
724	22	21	20	6	6	6	6	6	28	16
286	23	22	20	6	6	6	6	6	28	21
101.1	20	21	20	6	6	6	6	6	28	24
58	22	20	20	22	26	24	6	6	30	24
17G	20	21	18	6	6	6	6	6	30	22
18AF	24	23	22	30	40	30	6	6	30	28

[(b)] The antibiotic panel included (µg/disk): GEN, gentamicin (10); TOB, tobramycin (10); AMK, amikacin (30); NAL, nalidixic acid (30), CIP, ciprofloxacin (5); OFX, ofloxacin (5); SXT, sulfamethoxazole-trimethoprim (23.75-1.25); TMP, trimethoprim (5); FOS, fosfomycin (200); NI, nitrofurantoin (100).
